# The Promise and the Hope of Gene Therapy

**DOI:** 10.3389/fgeed.2021.618346

**Published:** 2021-03-24

**Authors:** Eleni Papanikolaou, Andreas Bosio

**Affiliations:** ^1^Department of Molecular Technologies and Stem Cell Therapy, Miltenyi Biotec, Bergisch Gladbach, Germany; ^2^Laboratory of Biology, School of Medicine, National and Kapodistrian University of Athens, Athens, Greece

**Keywords:** genome editing, hemopoietic stem cell, retroviral vectors, designer nucleases, CRISPR

## Abstract

It has been over 30 years since visionary scientists came up with the term “Gene Therapy,” suggesting that for certain indications, mostly monogenic diseases, substitution of the missing or mutated gene with the normal allele via gene addition could provide long-lasting therapeutic effect to the affected patients and consequently improve their quality of life. This notion has recently become a reality for certain diseases such as hemoglobinopathies and immunodeficiencies and other monogenic diseases. However, the therapeutic wave of gene therapies was not only applied in this context but was more broadly employed to treat cancer with the advent of CAR-T cell therapies. This review will summarize the gradual advent of gene therapies from bench to bedside with a main focus on hemopoietic stem cell gene therapy and genome editing and will provide some useful insights into the future of genetic therapies and their gradual integration in the everyday clinical practice.

## Introduction

The idea that a gene can be delivered into specific cell types and its expression can lead to therapeutic efficacy, dramatically improving the patients' quality of life, was originally introduced by Theodore Friedmann 45 years ago and was later strongly encouraged and realized by George Stamatoyannopoulos, one of the founding members of the American Society of Gene and Cell Therapy (ASGCT). In this setting, the drug, which in the case of gene therapy is a gene, is packaged within a vector used to facilitate its entrance into the patients' cells. Of course, the notion of gene therapy has evolved, and in general, we refer to gene therapy when a therapeutic process involves genetic manipulation of the patients' cells with the use of a nucleic acid. This is actually the most important difference between cell and gene therapy: in cell therapy, the cells are not genetically modified but instead are subjected to a certain manipulation involving cell culture and exposure to specific types of media whereas gene therapy is mediated by the addition of any nucleic acid. For obvious reasons, the idea of gene addition was particularly applicable in monogenic diseases based on the simplified notion of “adding the missing gene or the normal allele to compensate for the expression of the mutated allele.” However, under the view of the latest advancements, gene therapy does not correspond to an addition of a gene, otherwise missing in the patient's cells, but with a gene that could offer therapeutic benefit to the affected individual.

There are basically three types of gene therapy: *ex vivo, in vivo*, and *in situ*. In *ex vivo* gene therapy, the target cells are removed from the patient's body, engineered either by the addition of the therapeutic gene or by other genetic manipulations that allow correction of the phenotype of the disease. The “corrected” cells are subsequently re-infused to the patient. This type of intervention is also termed *in vitro* gene therapy and is particularly applicable to blood diseases: in the case of blood cancer, the target cell may be T and, most recently, NK cells, and the therapeutic gene is the chimeric antigen receptor (CAR). In the case of monogenic diseases, the target cell is the hemopoietic stem cell (HSC) and the transgene varies analogous to the disease. The viral vectors utilized in both cases are mostly retroviral vectors, belonging either in the lentiviral or the oncoretroviral families of *Retroviridae*. However, depending on the affected tissue, *ex vivo* gene therapy is not always the intended type of corrective approach. For example, if the target organ is the brain, the spinal canal, or the liver, another type of therapy is employed, termed *in vivo* gene therapy. In this setting, the therapeutic vector is administered systemically in the blood circulation or the cerebrospinal fluid of the patient, and depending on the disease, different types of viral vectors are utilized, such as adenoviral vectors (AVs) or adeno-associated viral vectors (AAVs). Finally, there is a last scheme of gene therapy, in which the viral vector is administered *in situ*, i.e., to a specific organ or area in the body of the patient either through direct injection, e.g., into the tumor (in the case of melanoma) or into suitable brain areas (in the case of neuropathies) or by an insertion of a catheter in the case that the organ to be treated is the heart. The selection of the procedure depends entirely on the type of indication, the affected tissue, and the cell type that requires correction. In contrast to HSCs, namely, CD34^+^ cells, that can be easily isolated from the patients, nerve stem cells are difficult to obtain for *ex vivo* manipulation. In addition, stem cells are only partially characterized in the liver. Hence, gene therapy for specific organs or indications is dependent on systemic or *in situ* administration of the therapeutic vector.

Although the idea of genome correction was quite innovative in its nature, especially during the 90s, clinical translation involving genome correction is still rare and adoption of the application of gene therapy at a wider scale and in the context of a medical routine has been only partial. To date, there are more than 2,600 clinical trials concerning gene therapy and/or genome editing, but very few therapeutic drugs have acquired marketing authorization for different indications (summarized in Table 1).

**Table 1:** Gene therapy products that have acquired marketing authorization.

**Name** **(Brand name)**	**Vendor**	**Indication**	**Type of indication**	**Approval region**	**Price (kE^**§**^)**
Onasemnogene abeparvovec (ZOLGENSMA^®^)	Novartis	Spinal muscular atrophy	Rare disease	2019 (USA)	2.125
Betibeglogene autotemcel (ZYNTEGLO^®^)	bluebird bio	Transfusion dependent β-thalassemia	Rare disease	2019 (EU)	1.575
Voretigene neparvovec (LUXTURNA^®^)	Spark Therapeutics	Leber's congenital amaurosis	Rare disease	2017 (USA)	850
Alipogene tiparvovec (GLYBERA^®^)	UniQure	Lipoprotein lipase deficiency	Rare disease	2012 (EU^*^)	1.000
STRIMVELIS^®^	Orchard Therapeutics	Severe combined immunodeficiency due to adenosine deaminase deficiency (ADA-SCID)	Rare disease	2016 (EU)	594
Tisagenlecleucel (KYMRIAH^®^)	Novartis	B acute lymphoblastic leukemia	Cancer	2017 (USA)	475
Axicabtagene ciloleucel (YESCARTA^®^)	Kite Pharma	Type of non-Hodgkin lymphoma	Cancer	2017 (USA)	373
Talimogene laherparepvec (IMLYGIC^®^)	Amgen Inc	Melanoma	Cancer	2015 (USA and EU)	65

* *Withdrawn in 2017, kE*

§ *thousands of euros*.

### Innovation

During the early times of its development, the gene therapy field has faced a lot of skepticism specifically after the unfortunate death of Jesse Gelsinger (Teichler Zallen, 2000) but also later on during the leukemic events recorded on the X-SCID clinical trial (Papanikolaou and Anagnou, 2010) in the early 2000s. The death of Jesse Gelsinger not only had a profound impact on the gene therapy field, it also underlined the general lack of knowledge about the vector–host interactions and ultimately pointed out the weak spots within the collaboration between the researchers and the regulatory agencies. Eventually, the case of Gelsinger has been quoted relatively recently for a number of times^1^ (Baker and Herzog, 2020) specifically in view of the coronavirus pandemic and the generation of a new and effective vaccine. Of note, one of these reports^1^ correlates the safety issues raised around the time of Gelsinger's death with the genome editing approaches currently employed, rather successfully, by a number of companies and academic institutions.

The scientific community is characterized by a heterogeneity in terms of taking risks, since there are scientists who intensely question the safety of any novel therapeutic approach and scientists who pave the way toward innovative and frequently risky treatments. A striking example of such risks and their potential to shape the policies around genetic therapies has recently happened in China, where the regulatory norms originally comprised mostly technical management methods or ethical guidelines under a broad legal framework issued by Commissions of the State Council in combination with departmental regulations and regulatory documents issued by individual ministries (Wang et al., 2020). It was only after the incidence with the CRISPR (Clustered Regularly Interspaced Short Palindromic Repeats) babies in November of 2018 (Lander et al., 2019) that urged China to advance legislation in areas of biosecurity, genetic technology, and biomedicine. To this end, the “Biosafety Law” was approved in 2019 by the Standing Committee of the National People's Congress. The aim of this law is to become a basic, systematic, comprehensive, and dominant legal framework on biosafety. Therefore, the regulatory landscape in genetic therapies is currently being shaped in China.

On the other hand, in Europe and in the USA, any new drug, regardless if it is gene therapy related, is not judged by the number or even the quality of publications, but eventually by the regulatory authorities who have the legal capacity to determine the marketing authorization of the formulation. However, the regulatory authorities have different views from the researchers in terms of innovation and safety. It is also important to keep in mind that regulators are basing their decisions on data and always compare those to the pre-existing state of the art of a specific indication in terms of equivalency. Hence, any new therapy, from a regulatory aspect will be thoroughly investigated and examined on the safety profile it presents and eventually on the extent of comparability between the currently authorized therapeutic treatments. This approach is employed both by the European Medicines Agency (EMA) in Europe and the Food and Drug Administration (FDA) in the United States (Iglesias-Lopez et al., 2020).

One strategy that can be utilized by regulators, including governments, health technology assessment (HTA) bodies, and health care decision makers, in order to advance and promote the development of novel medicinal treatments, is to recognize and award innovation. In the review of De Solà-Morales et al. (2018), the authors try to investigate how innovation is defined with respect to new medicines. Their conclusion is that innovation is differentially defined through countries, depending on independent political and societal factors. Hence, it is challenging to achieve common alignment, although coordination between countries and among regulators should be strongly encouraged as it would eventually help researchers and/or manufacturers toward determining mutually applicable research policies that can drive innovation. In their review (De Solà-Morales et al., 2018), components and dimensions of innovation are mentioned and include notions such as unmet need, health outcomes, novelty, step change, availability of existing treatments, efficacy, new molecular entity, molecular novelty, therapeutic value, market share, cost-saving, disease severity, clinical benefit, safety, pharmacological/technological differences from current treatments, etc.

Innovation in other industrial sectors is defined usually as any improvement of the end product either in terms of manufacturing or in terms of cost reduction in the long term. However, this usually does not apply in the health care sector: a new product is often substantially different from existing therapies and improvements in patients' quality of life; i.e., the therapeutic benefit, as a result of the application of the innovative approach, is of the greatest importance. Another major aspect is the overall expenditure associated with development of the novel approach by the health industry, which is usually high and is currently the focus of specific discussions in Europe and in the United States and ultimately points toward the affordability within the public health budgets (McCabe et al., 2009). However, as considerations about the costs are not usually included during the original design of the novel approach as a key component of innovation, there is the probability that innovation in pharmaceuticals and cell/gene therapy may not be aligned with the requirements of public or insurance health budgets and by extrapolation of society as a whole. Specifically for gene therapy approaches, the term “financial toxicity” is already circulating among the policy makers, the industry, and, consequently, the researchers.

Paradigms of definitions of innovations in different European countries are listed below: In France, the HAS (Haute Autorité de Santé) defines innovative products as those for which the producers assert a medium to major improvement of the clinical benefit compared to the currently available treatments [i.e., Amélioration du Service Médical Rendu (ASMR) of level I, II, or III] (O'Connor et al., 2016). Other agencies such as the Swedish Tandvårds–och läkemedelsförmånsverket (TLV), the Scottish Medicines Consortium (SMC), and the National Health Service in England (NHS) take into consideration the novelty of the approach but in combination with the improvement of patients' quality of life and any potential reformation of the health care system; i.e., the new therapeutic approach should present palpable added value (De Solà-Morales et al., 2018). Surprisingly, in the Netherlands, the Zorginstituut Nederland (ZINL) characterizes a product as innovative when it seems to be promising from a scientific point of view, but for which even insufficient data can overall provide a reasonably positive outlook and consequently effect a constructive response by the agency (De Solà-Morales et al., 2018). Finally in Germany, innovation is not referenced within the legal framework and in general the focus lies on the additional therapeutic benefits provided by the novel approach (De Solà-Morales et al., 2018).

To summarize, the definition of a novel medical approach as innovative in essence lies in its truly innovative nature. However, ideally, it should combine additional features such as (a) be at least as safe as the current treatments, (b) dramatically improve the patients' quality of life, and (c) be affordable by reimbursement bodies (payers). Finally, another important aspect would be to distinguish between price and the true value of the novel approach.

### Early Insights From Commercialization of Gene Therapies in Europe

Toward a better understanding of the impact that gene therapy presents at a societal level, one should keep in mind that in terms of innovation, any gene therapy approach is considered highly innovative. Consequently, in the context of genome editing, identification of the nucleases that generate targeted double strand DNA breaks that can, in a subsequent process, be repaired by indels or via homologous recombination and correct any genetic mutation was not only innovative but also considered a scientific breakthrough.

However, any marketing authorization of these products is expected to be scrutinized by the regulatory agencies as it was previously the case for other gene therapy products. Under the existing regulatory framework, cellular products that have been subjected to more-than-minimal manipulation are broadly classified as either medicinal products (EU) or biologics (USA). In Europe, cell-based medicinal products are regulated under the Advanced Therapy Medicinal Product (ATMP) Regulation, which mandates that all ATMPs are subject to a centralized marketing authorization procedure (Coopman, 2008). All marketing authorization applications are subject to a 210-day assessment procedure by the EMA, supported by the Committee for Advanced Therapies (CAT), before a license can be granted. Member states retain responsibility for authorization of clinical trials occurring within their borders and have the option to exempt certain products used on a non-routine basis for unmet clinical need, referred to as the “Hospital Exemption” based on Article 28 of Regulation (EC) 1394/2007. As with all medicines, the EMA continues to monitor the safety and efficacy of ATMPs after they are approved and marketed and provides scientific support to developers for designing pharmacovigilance and risk management strategies used to monitor the safety of these medicines.

Regulatory approval, however, does not guarantee availability to patients or reimbursement by European health systems, because novel therapies, regardless of their mechanism of action, have to undergo formal Health Technology Assessment (Touchot and Flume, 2017). From a time perspective, the first marketing authorization for gene therapy products for rare diseases occurred in 2012 with Glybera^®^ (EU), followed by Imlygic^®^ (EU and USA) in 2015 and Strimvelis^®^ (EU) in 2016. Therefore, these products have not only undergone meticulous evaluation from regulatory agencies, they have been also subjected to Health Technology Assessment by the reimbursement bodies and have received positive opinions from regulators and payers, and thus, a comprehensive analysis of their life cycle can now be conducted.

Glybera (alipogene tiparvovec) was the very first gene therapy agent to officially receive marketing authorization in Europe for treatment of lipoprotein lipase deficiency, a deadly disease causing severe pancreatitis to the affected patients. LPL deficiency (LPLD) is classified as a rare disease, estimated to occur in ~1 in 250,000 people in the general population and has been described in all races. Glybera was an adeno-associated serotype 1 vector (AAV-1), designed to deliver *in vivo* to the patients several copies of the normal allele (gene addition) by injection to several parts of the muscle areas of the body. Each vial of the vector had an estimated cost of ~100,000 euros, and to achieve a therapeutic quantity in the body of the patient, it was necessary to inject at least 10 vials. This fact raised the price of the therapy to 1 million euros. The drug was originally marketed by uniQure and, after going through formal evaluation through Health Technology Assessment in Germany and in France, failed to achieve a recognition of benefit in either country (Touchot and Flume, 2017). In France, the HAS Transparency Commission stated that (Touchot and Flume, 2017):

➢ “A moderate effect on triglycerides and on episodes of pancreatitis has been observed but this effect was not sustained in the medium–and long-term” (in line with submitted efficacy data showing only transient efficacy);
➢ “The clinical relevance of the chosen primary efficacy endpoint (reduction in the triglyceride level) is debatable;”
➢ “Uncertainties about the short–and medium-term safety of this gene therapy, which cannot be re-administered because of its action mechanism, remain.”

As a result, the HAS concluded that the actual benefit of Glybera is insufficient to justify reimbursement by the French national health insurance and thus the product was not commercialized in France.

In Germany, it was initially assessed as a community product but was evaluated by AMNOG (the German Health Technology Assessment process) to confer “unquantifiable additional benefit” because of lack of proper clinical data that would adequately justify the actual therapeutic potency of the product (Touchot and Flume, 2017). This led to a repositioning of the drug to a hospital-only product and allowed price negotiations directly between hospitals and payers. In the case of Germany, these discussions were fruitful only for a single patient that was treated at Charité in Berlin in September 2015 with an estimated price of 900,000 euros after an agreement with DAK (Deutschen Angestellten-Krankenkasse), a large German health insurance provider. This patient, was a woman with LPLD who suffered consecutive debilitating pancreatitis and was hospitalized in intensive care more than 40 times, and thus, she qualified for gene therapy because of the severity of her overall clinical status. The woman was fully cured and never suffered from pancreatitis again (Crowe, 2018). Despite these hopeful events and taking into account the very low number of patients, uniQure decided in 2015 not to apply for approval in the USA and exclusively licensed rights in Europe to Chiesi Farmaceuticals for €31 million (Regalado, 2016). A total of three remaining doses left on the shelf were basically given away in one patient from Italy and two German patients who received doses for 1 euro each. Since October 2017, the utilization of Glybera was discontinued in EU because marketing authorization to Chiesi Farmaceuticals was not renewed, for financial reasons.

Imlygic (Talimogene laherparepvec), which has been authorized for treatment of melanoma, is a vector based on a strain of Herpes Simplex Virus 1 (HSV-1) that possesses oncolytic properties in combination with the expression of granulocyte-macrophage colony-stimulating factor (GM-CSF) to attract antigen-presenting cells (APCs) in the affected area. Upon administration *in situ*, Imlygic lyses tumor cells, enhances antigen loading of MHC class I molecules, and express GM-CSF to increase tumor antigen presentation by dendritic cells (Conry et al., 2018). Therefore, although it is administered *in situ*, it provokes a systemic anti-tumor immunity. It was approved by the EMA and FDA in October and December of 2018, respectively (Touchot and Flume, 2017). Upon approval of the regulatory agencies, evaluation of Imlygic has been completed so far in the UK (Touchot and Flume, 2017). Initially, the NICE (National Institute of Clinical Excellence) concluded that Imlygic, despite its truly innovative mechanism of action, was not cost-effective and did not confer significant advantage in terms of the overall survival of the patients compared to the existing therapies for melanoma. This evaluation prompted the company to discuss a respective discount with the Department of Health (Touchot and Flume, 2017), to agree to a patient access scheme, and to narrow the indication of coverage to patients who did not qualify for systemically administered immunotherapies. Imlygic is currently still being evaluated in Germany by IQWiG (the German health technology assessment body) and the Federal Joint Committee (G-BA), which requested additional data to complete the assessment including comparison with administration of GM-CSF alone. Of note, previously in clinical trials, the overall response rate (ORR) was increased in the Imlygic arm (26.4%) compared to the GM-CSF arm (5.7%). The mean overall survival (OS) was 23.3 months in the Imlygic arm, vs. 18.9 months on the GM-CSF arm (*p* = 0.051), showing a marginal statistical trend in favor of Imlygic (Andtbacka et al., 2015). However, administration of GM-CSF is also not an authorized treatment for melanoma. This poses a risk toward the final positive evaluation of Imlygic as it could be again classified as providing “no quantifiable additional benefit,” suggesting that it is probable that it will face challenges in reaching a wider number of patients, unless newly generated data provide an undisputable therapeutic benefit compared to the standard treatment, as this is defined by each individual payer (Touchot and Flume, 2017).

The aforementioned products are employed in *in vivo* and *in situ* gene therapy, respectively. However, one of the greatest achievements in the history of the field was the case of Strimvelis^®^. Strimvelis^®^, is a product derived from genetic engineering of HSCs isolated from patients suffering from severe combined immunodeficiency due to adenosine deaminase deficiency (ADA-SCID). In this case, genetic correction of HSCs is mediated by gene addition of the normal allele packaged inside an oncoretroviral (also termed gamma retroviral) vector. In terms of safety, the gene therapy field has been severely hampered by the unfortunate leukemic events that occurred during the clinical trial for another form of SCID, namely, the X-SCID. In the early 2000s, four cases of leukemia in the French X-SCID clinical trial were recorded out of the initial seven infants that were recruited for the study. These events were attributed to the vector's integration into the proto-oncogene LMO2 (Hacein-Bey-Abina et al., 2003a; Kohn et al., 2003) and triggered a new field of research resulting in a comprehensive characterization of the preference to integrate of lentiviral vectors and oncoretroviral vectors (Montini et al., 2006; Biasco et al., 2017) within the human genome. Surprisingly, although lymphoproliferative aberrations were also observed in the trials of HSC gene therapy for Wiskott–Aldrich syndrome (Braun et al., 2014) and for chronic granulomatous disease (CGD, Stein et al., 2010), no case of leukemic events for ADA-SCID in the context of clinical trials has been recorded, despite the fact that all the aforementioned indications employed oncoretroviral vectors. Unfortunately, 4 years after Strimvelis^®^ received marketing authorization, lymphoid T cell leukemia has been reported in one patient in October of 2020, and its relationship to the gene therapy is currently under investigation (Ferrari G. et al., 2020). Strimvelis^®^ was originally developed in Ospedale San Raffaele in Milan (Aiuti et al., 2002, 2009) in collaboration with Fondazione Telethon before it was acquired by GlaxoSmithKline and, in May 2016, received approval in Europe. GSK initially collaborated with MolMed a clinical biotech company, to develop a robust process for commercializing the product. Because Strimvelis^®^ contains essentially HSCs that need to be engineered within a very short period of time (not more than 2 days), until today, it was authorized only in Italy (MolMed) and patients from other European countries are supposed to travel to Italy to receive the treatment (Touchot and Flume, 2017). The Italian medicines agency (AIFA) agreed to a reimbursement of 594,000 euros based on the substantial clinical benefit for the patients in combination with the overall amount spared from a lifetime treatment with enzyme replacement therapy, as Strimvelis was beneficial for the public health budget in the long run (Touchot and Flume, 2017). In 2018, GSK transferred all the assets associated with Strimvelis to Orchard Therapeutics (Paton, 2018). Although the product has to undergo evaluation also in other European countries, and despite the small number of patients treated so far, it should be mentioned that the short time period between the approval and the reimbursement decision by the Italian authorities indicates that good clinical practice, good manufacturing practice, and robust clinical data combined with reasonable pricing can pave the way toward integrating gene therapies in medical routine. Of course, the report of the leukemic event is expected to create delays toward authorization in other countries until the results of the investigations are announced.

Last but not least, another important achievement for HSC gene therapy is Zynteglo^®^, which received marketing authorization for treatment of transfusion dependent β-thalassemia (TDT), a disease that was the first candidate for HSC gene therapy. Significant research efforts toward the generation of erythroid-specific globin expressing lentiviral vectors were employed that were eventually successfully translated to clinical trials in 2006 (Ferrari G. et al., 2020). Zynteglo^®^, similar to Strimvelis^®^, is a product derived from genetic engineering of HSCs isolated from patients suffering from TDT, transduced with BB305 lentiviral vector, which encodes a β-globin transgene (βT87Q globin), which also has antisickling properties. The results of phase I and phase II trials were reported and showed that gene therapy was efficacious in 80% of patients with non-β^0^/β^0^ genotypes and 38% of patients with β^0^/β^0^ genotypes, measured by transfusion independence at the 2-year follow-up (Ferrari G. et al., 2020), while the rest of the participants reached various levels of transfusion reduction. On the basis of these results, Zynteglo^®^ received conditional marketing authorization for use in patients with transfusion-dependent β-thalassemia with non-β^0^/β^0^ genotypes in 2019 in Europe, while the respective authorization by the FDA is still pending.

Undisputable success stories in the field of CAR-T gene therapy are also Kymriah and Yescarta. However, Zynteglo^®^, Kymriah^®^, and Yescarta^®^ have relatively recently received regulatory approval, and their assessment in terms of reimbursement is currently ongoing in EU and USA.

### Excellent Science and Safety

Gene therapy based on viral vectors utilizes the natural ability of viruses to deliver genetic material to cells, and a large part of research has been devoted toward generating novel, more efficient, and safer delivery tools employing gammaretroviruses, lentiviruses, adenoviruses, and adeno-associated viruses. Retroviruses are particularly applicable in the case of HSC gene therapy because they have the unique capability to fully integrate their genome intact into the genome of the host cell. However, as with any new therapeutic approach, gene transfer using viral vectors also introduced new side effects. One of these side effects, known as insertional mutagenesis or genotoxicity, involves activation of proto-oncogenes or disruption of tumor suppressor genes due to retroviral vector integration. Of course, genotoxicity is a natural phenomenon that has been described since the discovery of retrotransposons, as transpositions of Long Interspersed Nuclear Elements (LINEs) were (i) detected as *de novo* insertions into the coding regions of factor VIII gene resulting in hemophilia A, (ii) integrated into the adenomatous polyposis coli tumor suppressor gene causing its disruption and generating colon cancer, (iii) detected into the myc locus in a breast cancer, and (iv) inserted into exon 48 of the dystrophin gene (Löwer et al., 1996). These transpositions were detected in extremely very low frequency within the overall population and even within the population suffering from these specific indications. Regarding the utilization of retroviral vectors into gene therapy protocols, although the possibility of insertional mutagenesis was originally discussed as theoretically possible, such risks had been estimated to be extremely low, based on (a) the fact that over 90% of human genome is non-coding and (b) the assumption that proviral integration into the human genome would be random (Papanikolaou et al., 2015). Unfortunately, these hypotheses were not verified after the reports of lymphoproliferation due to insertional activation of the LMO2 gene following gene therapy in the French X-SCID clinical trial (Hacein-Bey-Abina et al., 2003a,b), the leukemias developed in the Wiskott–Aldrich gene therapy trial (Braun et al., 2014), and myelodysplasia attributed to EVI1 activation after gene therapy for CGD (Stein et al., 2010). All these events highlighted the importance of understanding the underlying mechanisms that are responsible for integration into the preferred genomic loci but also the components that contribute toward the repair of the genome during the integration events. From a phenotypic standpoint, this lack of knowledge was translated as leukemic events only during clinical trials, as such events were not detectable during the pre-clinical development of gene therapies of the aforementioned indications. From a regulatory standpoint, the clonal dominance observed during the French β-thalassemia trial (Cavazzana-Calvo et al., 2010) led to a clinical hold of the specific trial as per FDA guidelines for 5 years, until it was clear that the respective clonal dominance did not evolve to any kind of dysplasia or leukemia and it was safe to proceed and recruit more patients to the study.

All the aforementioned cases underlined the non-random integration patterns of retroviral vectors and sparked the field's interest toward characterizing the potential mechanisms. Therefore, it was comprehensively shown that gammaretroviral vectors preferentially locate around transcription start sites while HIV-based vectors strongly favor integration in transcriptional units and gene-dense regions of the human genome (Papanikolaou et al., 2015). These properties rendered lentiviral vectors safer for gene therapy approaches compared to oncoretroviral vectors and paved the way toward substitution of oncoretroviral by lentiviral vectors. Indeed, lentiviral vector-based gene transfer into HSCs has subsequently been applied in the treatment of X-linked adrenoleukodystrophy (Cartier et al., 2009), metachromatic leukodystrophy (Biffi et al., 2013; Sessa et al., 2016), and Wiskott–Aldrich syndrome (Aiuti et al., 2013) without any vector-related adverse events. Therefore, the clonal dominance observed in the β-thalassemia trial is still an open question regarding whether this was purely coincidental or was truly attributable to a clonal proliferation as a result of the HMGA-2 dysregulation.

Aside from the comprehensive characterization of the integration preference of onco- and lentiviral vectors, the field furthermore strengthened the efforts toward making gene therapy safer by generation of self-inactivating (SIN) vectors. Because activation of the LMO2 oncogene was attributed to the strong enhancer elements within the U3 region of the retroviral Long Terminal Repeats (LTRs) (Hacein-Bey-Abina et al., 2003a), part of the U3 enhancer was removed in order to minimize the probability of activating neighboring oncogenes. In addition, alternative genetic elements, such as chromatin insulators, were gradually incorporated in the remaining U3 region of the LTR. Chromatin insulators are DNA sequences capable of maintaining the expression of a gene region independently of the expression of the neighboring gene region, by inhibiting their natural interactions (insulation). Insulator sequences have two main characteristics: (a) barrier activity, i.e., gene expression of a chromatin region is not affected by the adjacent heterochromatin region if an insulator is inserted between them, and (b) enhancer blocking activity, i.e., inhibition of the concerted action between a promoter and an adjacent enhancer (Heger and Wiehe, 2014). Therefore, the incorporation of a chromatin insulator into the U3 region of the LTR, on the one hand, offers additional protection against the activation of neighboring oncogenes and, on the other hand, ensures the expression of the therapeutic gene in case of integration in a heterochromatic region. For globin gene therapy, significant efforts have been employed to this end due to long-standing knowledge that the expression of globin genes was variable due to the integration of the vector into transcriptionally inactive regions of chromatin, i.e., dependent on “position effects” (Persons et al., 2003). Additional efforts deriving from the group of Dr. Stamatoyannopoulos have demonstrated the need to incorporate chromatin insulators into vectors intended for the gene therapy of hemoglobinopathies (Aker et al., 2007). However, later studies showed that incorporation of chromatin insulators leads to a significant loss of titer of the lentiviral vector (Urbinati et al., 2009), which typically translates to greater manufacturing costs as more vector is necessary to achieve the ideal transduction efficiency that would suffice to exhibit therapeutic efficacy. Currently, in the ongoing clinical trials of bluebird bio, the globin vector utilized is insulator-free (Negre et al., 2015), and as previously stated, it remains unclear whether the initial clonal dominance was because of the higher proliferation rate of a specific clone as a result of the vector integration into the HMGA gene or whether this observation merely reflects the effects of incorporating a limited number of genetically modified hematopoietic stem cells into the patient's marrow. Thus, bluebird's vector format is not considered dangerous from a regulatory standpoint.

Excluding genotoxicity, in clinical protocols that utilize lentiviral vectors, the regulatory agencies are also concerned about recombination events that might occur during the manufacturing process of the vectors and require extensive data demonstrating the lack of replication-competent retroviruses or lentiviruses (RCRs/RCLs) partly because the agencies assume higher probability for genotoxicity if RCRs or RCLs are present (Milone and O'Doherty, 2018). In addition, they request long-term follow-up monitoring of the patients participating in cell and gene therapy studies for the presence or RCRs/RCLs, new incidence or re-appearance of autoimmune, rheumatologic, and neurological disorders, or delayed malignancies, as a result of genotoxicity. Toward generating safer tools to reduce the risk of insertional mutagenesis, integration-deficient lentiviral vectors (IDLVs) or non-integrating lentiviral vectors (NILVs) have been generated (Wanisch and Yáñez-Muñoz, 2009; Milone and O'Doherty, 2018), which present lower probability of causing either genotoxicity or generating RCRs. Unfortunately, their use is rather limited because they provide merely transient transgene expression in proliferating cells, but they can still be employed to promote stable expression in non-dividing cells or to induce RNA interference and mediate homologous recombination (Wanisch and Yáñez-Muñoz, 2009).

To summarize, clinical trials in gene therapy via gene addition were initiated in the early 1990s, and until the late 2010s, a significant amount of effort combining excellent science and extensive assessment of potential risk factors have managed to make gene therapy more robust and simultaneously achieve great advancements toward clinical benefit.

## The Era of Genome Editing: Challenges and Prospects

### Prospects

Over the last decade, the discovery of important novel regulatory elements of the human genome, combined with the continuous developments of novel technologies in the field of molecular biology and biotechnology, has conferred important conceptual insights for the implementation of new molecular approaches for the treatment of monogenic disorders. The advent of induced pluripotent stem cells and the design of novel nucleases that target specific areas in the genome have rendered gene editing approaches pivotal players in the field of therapy of inherited diseases. Gene targeting that is currently mediated by genome editing, is anticipated to outperform the classical approach of gene therapy via gene addition utilizing retroviral vectors, mainly due to the inability of the latter to establish targeted vector integration into the host genome.

Gene editing technology allows site-specific genome modifications, ranging from single-nucleotide edits to large deletions/inversions or targeted integration of entire genes, and is anticipated to outperform the classical approach of gene therapy via gene addition utilizing retroviral vectors, in part due to the inherent risk of insertional mutagenesis of gene addition by retroviral vectors and its limitations to treat gain-of-function mutations or defects in large genes. Moreover, in contrast to gene addition, most gene editing approaches maintain the natural genomic regulation of the gene of interest and thus physiological expression.

The original and still most prevalent application of gene editing for therapy relies on double strand breaks in DNA, which are introduced by engineered nucleases that act at predetermined and targeted genomic loci (Genovese et al., 2014). Such nucleases are:

- Zinc Finger Nucleases (ZFNs)
- Transcription Activator-Like Effector Nucleases (TALENs)
- Cas nuclease of the CRISPR/Cas9 system.

Their mode of action is to induce a double strand break (DSB) on the DNA molecule followed by respective repair either through the non-homologous end joining (NHEJ) or via homologous recombination (HR). Through NHEJ, repair of the DSBs leads to disruption of the target sequence by generation of small insertions or deletions, which collectively are called “indels.” Repair through HR leads to full reconstitution of the target sequence if a template donating a homologous sequence, that serves as a matrix for the repair to take place, is provided.

It should be noted, however, that a DSB is actually the initiating step in natural genome editing and occurs in mammalian cells on several occasions, such as the V(D)J recombination through the RAG1/RAG2 enzymes (Jasin and Rothstein, 2013), during the meiotic recombination mediated by the Spo11 nuclease (Jasin and Rothstein, 2013) and finally during the natural gene drives, managed by homing endonucleases (Burt, 2003). Also, all mammalian cells possess robust DNA repair mechanisms; however, the frequency of repair either through NHEJ or HR increases at least by a 100-fold following a double strand break (Jasin and Rothstein, 2013). Therefore, the novel engineered nucleases are necessary to achieve adequate gene correction to reach the anticipated therapeutic levels required. This aspect is of particular interest for the clinical applications of engineered HSCs, because the number of CD34^+^ cells that need to be infused to patients are in the range of 5 × 10^6^-10^7^ cells/kg and 80% of those should be genetically corrected. For example, for a thalassemic patient with an average weight of 70 kg, one would need to infuse 5 × 10^6^ × 70, i.e., a total of 3.5 × 10^8^ viable CD34^+^ cells, of which at least 80% should be genetically corrected. Thus, in order to have a final total cell count of ~4–5 × 10^8^ cells in the final cell product, it is anticipated that optimization toward mobilization of HSCs to the periphery specifically from patients suffering from rare diseases, optimization of infusion protocols, as well as optimization of the editing process *per se* are absolutely necessary. These are current challenges that will increasingly appear as we pave the way toward clinical genome editing applications. For example, even optimized transfer of nucleases by electroporation leads to a significant loss of cell viability, which, in turn, necessitates efficient mobilization and collection of high numbers of HSCs as editing substrate. Unfortunately, because in certain cases, such as in the case of sickle cell disease, patients mobilize poorly or due to innate characteristics of the disease *per se* use of granulocyte colony stimulating factor (G-CSF) is not recommended, one of the first challenges toward clinical translation would be the existence of a validated freezing protocol followed by a validated thawing protocol as it is possible that certain patients would need to undergo multiple rounds of mobilization.

A second notable challenge is the process of genome editing in terms of culture conditions including media, cytokines, timelines, and inclusion of several means of molecules or strategies to enhance the efficiency of the editing. To this end, several amendments have been published. Dever et al. (2016) reported a CRISPR/Cas9-based gene editing approach that combines Cas9 ribonucleoproteins (RNPs) and delivery of a homologous template via an AAV to achieve homologous recombination at the β-globin gene in HSCs combined with a concomitant purification method that generates a population of hemopoietic stem and progenitor cells with more than 90% targeted integration. Respective results were also obtained for SCID-X1 (Pavel-Dinu et al., 2019) following the same approach, i.e., the CRISPR/Cas9-AAV6-based strategy to insert the cDNA of the normal gene into the endogenous start codon. This approach aims to functionally correct disease-causing mutations throughout the genomic locus. Unfortunately, a similar strategy could not be employed for hemoglobinopathies as the presence of genomic introns is mandatory to achieve tissue specificity as well as therapeutic expression levels (Uchida et al., 2019).

Another interesting approach to achieve higher editing efficiency is to modulate the cellular pathways responsible for DSB repair. More specifically, the efficiency of HR by genome editing is limited by DSB repair pathways that compete with homology-directed repair (HDR), such as non-homologous end joining (NHEJ) (Nambiar et al., 2019). The choice of the type of the DSB repair pathway is mostly determined by the DSB resection, a nucleolytic process that converts DSB ends into 3′-single-stranded DNA overhangs (Nambiar et al., 2019). Certain NHEJ factors, including 53BP1, promote the direct joining of DSBs by protecting DNA ends from resection. Limited resection of DSB ends can expose regions of sequence microhomology, which favor DSB repair through microhomology-mediated end joining (MMEJ), while more extensive DSB resection generates the long 3′-single-stranded DNA tails required for HDR (Nambiar et al., 2019). Thus, cellular factors that impede DSB resection represent major barriers to HDR-mediated precision genome editing. Toward this direction, the authors characterized RAD18 as a stimulator of CRISPR-mediated HDR and identified its mechanism of action that involved suppression of the localization of the NHEJ-promoting factor 53BP1 to DSBs (Nambiar et al., 2019).

An alternative strategy to enhance the efficiency of genome editing was to transiently silence p53 (Schiroli et al., 2019). More specifically, Schiroli et al. challenged the successful use of the combination of AAV and generation of DSBs by engineered nucleases such as ZFNs and CRISPR/Cas9 by claiming that they cause excessive DNA damage response (DDR) across all hemopoietic stem and progenitor cell subtypes analyzed (Schiroli et al., 2019). DDR consequently induced cumulative p53 pathway activation, constraining proliferation, yield, and engraftment of edited HSPCs, which could be overcome by transient inactivation of p53. Of note, DDR is reported to be activated also under conditions of viral infections or vector transduction as there are recent reports correlating immune responses within the cells that undergo DNA damage (Piras et al., 2017; Dunphy et al., 2018). Immune responses have also been detected in the context of gene therapy via gene addition (Papanikolaou et al., 2015) after transduction of CD34^+^ cells with a GFP encoding lentiviral vector. It is not unprecedent that such immune responses are linked to DNA damage repair mechanisms, since retroviral integration presupposes breaks on the DNA chain. However, it should be noted that DDR is not always activated: For example, in the study by Papanikolaou et al. (2015), transduction with the GFP lentiviral vector activated immune responses without significant DDR. On the contrary, in the study by Piras et al. (2017), there was significant upregulation of DDR. One important difference between the two studies was the multiplicity of infection (MOI); in the first study, an MOI = 10 was employed, while in the second study, the authors experimented with MOI = 100. These results immediately suggest that the MOI plays a crucial role during the manufacturing process since both studies employed a VSV-G pseudotyped GFP encoding lentiviral vector and used cord blood CD34^+^ cells. Obviously, a better understanding of the interplay between vectors or nucleic acid molecules with the host cell in terms of both quality and quantity would be necessary to advance the field of gene engineering. An important aspect that is linked to clinical translation is that activation of vector-mediated DDR can induce significant, albeit mild, increase in apoptosis of human HSCs in culture (Piras et al., 2017), which typically results in lower engraftment of engineered HSCs *in vivo*, particularly during the early phases of hemopoietic reconstitution (Piras et al., 2017; Piras and Kajaste-Rudnitski, 2020). Therefore, induction of DDR mechanisms in the context of genome editing should be taken into serious consideration, and strategies toward achieving robust and efficient editing without interfering with the stem cell-like character of CD34^+^ should be generated.

As reviewed by Piras and Kajaste-Rudnitski (2020), HSCs have devised several strategies of responding to RNA molecules as well as ssDNA and dsDNA molecules. Indeed, a plausible approach to increase the efficiency of retroviral transduction or gene editing would be to assess the mechanisms of innate immunity and nucleic acid sensing in HSCs and harness their potential. For example, transient silencing of cellular nucleic acid sensors could increase the level of transduction or the efficiency of the editing. To that end, many researchers have focused on several transduction enhancers such as 16,16-dimethyl prostaglandin E2 (PGE2) and LentiBOOST™, poloxamers, the polycationic protamine sulfate, cyclosporine A and cyclosporine H, and rapamycin (Piras and Kajaste-Rudnitski, 2020). PGE2 and LentiBOOST™ are already employed in the context of clinical trials (Tisdale John et al., 2018), but it should be emphasized that the exact mechanism of action of the majority of these transduction enhancers is not fully elucidated. Besides the employment of transduction enhancers, additional strategies exist in terms of culture conditions that urge HSCs to move toward the S phase of the cell cycle in order to increase the successful HR. One strategy employed by Ferrari S. et al. (2020) was to transiently downregulate p53 with GSE56 in addition to including the E4orf6/7 protein of adenovirus, a known interactor with cellular components involved in survival and cell cycle (Ferrari S. et al., 2020) to successfully enhance the efficiency of editing. From another perspective toward advancing safety, Wiebking et al. (2020) disrupted the uridine monophosphate synthetase (UMPS) involved in the pyrimidine *de novo* synthesis pathway rendering proliferation dependent on external uridine and providing thus the possibility to control cell growth by modulating the uridine supply. However, it should be noted that disruption of UMPS would be an additional genome editing process on top of any other correction, suggesting that to manufacture cell products that have been genetically engineered and present advanced safety features, one would have to edit at least two genomic loci. Although both strategies (Ferrari S. et al., 2020; Wiebking et al., 2020) certainly assume great potential, they involve genetic manipulation beyond the current state of the art, and the transition to the clinic will probably be challenging from a regulatory standpoint.

A final aspect of great importance is the type of mutations that are introduced in the human genome in the context of therapy. One idea would be to add the desired transgene into a safe harbor. Papapetrou et al. (2011) characterized as safe harbors specific genomic loci based on their position relative to contiguous coding genes, microRNAs, and ultraconserved regions. Genomic safe harbors should fulfill the following criteria: (i) distance of at least 50 kb from the 5′ end of any mapped gene, (ii) distance of at least 300 kb from any cancer-related gene, (iii) distance of at least 300 kb from any microRNA (miRNA), (iv) location outside a transcription unit, and (v) location outside ultraconserved regions (UCRs) of the human genome (i.e., enhancers, exons, regulatory sequences, etc.). The idea is promising and has been widely employed in the context of induced pluripotent stem cells, and most lately, it was capitalized by Gomez-Ospina et al. (2019) toward showing therapeutic benefit for Mucopolysaccharidosis type I by generating a CRISPR/Cas9 approach that targets the lysosomal enzyme iduronidase to the CCR5 safe harbor locus in human CD34^+^ hematopoietic stem and progenitor cells. The authors demonstrated adequate therapeutic efficacy in an immunocompromised mouse model of Mucopolysaccharidosis type I and showed that the modified cells could secrete supra-endogenous enzyme levels, maintain long-term repopulation and multi-lineage differentiation potential, and provide biochemical and phenotypic improvement *in vivo*.

Therefore, one approach is to introduce the therapeutic transgene into a safe harbor locus. Another approach is to introduce the therapeutic gene exactly in its natural position in the genome, thus ensuring lifelong regulation by the naturally occurring expression modulating elements affecting the respective region. This was already described to treat X-SCID 1 (Pavel-Dinu et al., 2019) but is a particularly plausible approach for hemoglobinopathies aiming to correct either mutations within the β-globin gene in the case of β-thalassemia, or the specific point mutation for sickle cell disease. To that end, at least two successful strategies have been developed aiming to correct the IVS I-110 (G>A) mutation in β-thalassemia (Patsali et al., 2019) via either CRISPR/Cas9 or TALENS or the sickle cell mutation (Park et al., 2019). However, the most widely employed approach applicable for both sickle cell disease and thalassemia is the induction of fetal hemoglobin via genome editing. In 2013, the group of Stewart Orkin mapped a regulator of expression of BCL11A specific for the erythroid lineage (Bauer et al., 2013), and a follow-up study employing genome editing proved that targeted disruption of the critical GATA1 binding motif within the +58 intronic BCL11A enhancer leads to indel generation and thereby to reduced BCL11A expression with associated induction of γ-globin expression in erythroid cells (Wu et al., 2019). This notion was moved to the clinic by two ongoing clinical trials, NCT03745287 by CRISPR Therapeutics and NCT03653247 by Bioverativ. The two trials differ in the designer nucleases used to target the enhancer in that CRISPR Therapeutics utilizes a CRISPR approach, while Bioverative utilizes a ZFN. Regarding the CRISPR trial, short-term results of 15–18 months of follow-up reported two patients, one with thalassemia and a second with sickle cell disease, who demonstrated significant increase in hemoglobin values (expressed in g/dl) after gene therapy, combined with the presence of over 95% F-cells in peripheral blood (Frangoul et al., 2020). This recapitulation of the HPFH (Hereditary Persistence of Fetal Hemoglobin) phenotype has become a common approach and was also employed as a therapeutic alternative by other researchers as well, first by disrupting the BCL11A binding motifs in the promoters of γ-globin genes by CRISPR (Métais et al., 2019) or TALENs (Lux et al., 2018), so as to inhibit the binding of BCL11A and hence prevent the silencing of γ-globin and also by comparing disruption of different HbF repressors, including KLF1 (Lamsfus-Calle et al., 2020) and LRF (Weber et al., 2020). Finally, efforts to reconstitute naturally occurring deletions that lead to loss of putative silencers located at the 3′ end of the γ-globin genes have been employed, including the 7.2-kb “Corfu” deletion of the γ-δ intergenic region and the 13.6-kb deletion including the γ-δ intergenic region and extending to the first intron of the β-globin gene, similar to the “Sicilian” 12.9-kb HPFH-5 deletion (Lattanzi et al., 2019).

Last but not least, another promising option is base editing by nucleotide deaminases linked to programmable DNA-binding proteins. These proteins function by fusing inactive or nickase Cas9 to deaminases that catalyze the enzymatic conversion of C to T (G-to-A on the opposing strand) or A to G (T-to-C on the opposing strand) (Gaudelli et al., 2017). Because this approach does not involve generation of DNA double strand breaks, it is supposedly safer compared to “classical” genome editing; however, certain limitations exist, as the currently available range of base editors cannot enable conversion of the sickle cell mutation, i.e., direct T-to-A correction. Nevertheless, the strategy can be employed to disrupt alternative sequence elements, analogous to NHEJ-mediated methods, to correct specific mutations of β-thalassemia (Zeng et al., 2020). Subsequent work by Liu and co-workers led to the concept of prime editing, which improved upon the versatility of their base editing tools by inclusion in the RNP particle of a reverse transcriptase and a template for reverse transcription. The resulting tools can precisely introduce all conceivable 12 nucleotide changes as well as small indels (Anzalone et al., 2019). Of note, an extremely interesting study was published in 2016 by Bahal et al. (2016) introducing the use of triplex-forming peptide nucleic acids (PNAs). PNAs are designed in a way that permits their binding to specific genomic DNA sites via strand invasion and formation of PNA/DNA/PNA triplexes (via both Watson–Crick and Hoogsteen binding) with a displaced DNA strand. PNAs are essentially nanoparticles consisting of a charge-neutral peptide-like backbone and nucleobases, enabling hybridization with DNA with high affinity. These PNA/DNA/PNA triplexes are potent in recruiting the cell's endogenous DNA repair systems to initiate site-specific modification of the genome when single-stranded “donor DNAs” are co-delivered as templates containing the desired sequence modifications (Anzalone et al., 2019). The results of this study proved the efficacy of nanoparticles in terms of phenotype correction in the context of monogenic diseases.

### Challenges

Undoubtedly, the research regarding all potential applications in the field of genome editing is very promising and perhaps has better long-term prospects compared to gene therapy by retroviral vectors. Gene addition by designer nucleases outperforms the classical gene addition by retroviral vectors because it provides targeted integration, which, so far, cannot be achieved with retroviral vectors. However, despite potentially higher safety, caveats still exist for genome editing.

The first very important challenge in terms of safety is the identification of the off-target effects. To that end, major efforts have been described including Digenome-seq (Kim et al., 2015) and CIRCLE-seq (Tsai et al., 2017; Lazzarotto et al., 2018). Both methods are based on adapter ligation to the CRISPR generated ends: Digenome-seq generates *in vitro* Cas9-digested whole-genome fragments and then proceeds to profile genome-wide Cas9 off-target effects in human cells. CIRCLE-seq generates a library of circularized genomic DNA with minimized numbers of free ends and subsequent treatment of purified circles with CRISPR/Cas9 RNP complexes followed by adapter ligation and high-throughput sequencing. Although both approaches are highly promising, there are limiting steps such as the length of reads during NGS. Additional efforts such as BLISS (Yan et al., 2017) involve fixation of cells and it is doubtful if there is high accuracy in introducing DSBs as part of the screening (and not the therapeutic) process at high accuracy. Finally the DISCOVER-SEQ (Wienert et al., 2019) approach is based on recruitment of specific DNA repair proteins; hence, it is questionable if all DSBs can be identified, given the fact that even the amount of the engineering agent can have a profound impact on the same cell type: For example, there have been differences described between engineered cord blood CD34^+^ by lentiviral vectors with low MOI (Papanikolaou et al., 2015) compared to high MOI (Piras et al., 2017). Excluding the actual limitations existing in the current approaches, another point of concern is the fact that some off-targets may be completely benign, whereas others could have serious consequences depending on the cell context or the indication. This is a well-recognized issue in the field and is currently being addressed by engineering the CRISPR payload at both the protein and gRNA level with simultaneous optimization of the ideal window of active exposure of the cells of interest to the functional RNP complex (Tay et al., 2020).

Therefore, the burden from a regulatory aspect is major for the following reasons: (a) Even a single genetic disease caused by knockout of a single gene or sequence may be associated with several mutations, even unrelated ones, in different patients. For example, nobody knows or can accurately predict what can be caused by disruption of the erythroid specific enhancer within the second intron of BCL11A at a population scale. (b) Depending on the indication, even the most well-characterized agents in the field of gene therapy still present surprises. The latest manifestation of tumor generation after lentiviral mediated gene addition in the context of CGD is alarming (Jofra Hernández et al., 2020), as the authors described the development of T cell lymphoblastic lymphoma and myeloid leukemia in 2.94% and 5.88% of the mice tested, respectively, and oligoclonal composition with rare dominant clones harboring vector insertions near oncogenes in these mice. (c) Genetic engineering of HSCs presents additional hurdles as CD34^+^ cells are difficult to be tested for karyotypic analysis, as most of the cells reside in Go phase. This poses a certain challenge toward identification of large chromosomal rearrangements as a result of designer nuclease action in the patients' genome, suggesting the need for development of surrogate assays. For example, approaches introducing chromosomal deletions and not indels will most probably face several difficulties during the transition toward a clinical trial. (d) Last but not least, gene therapy products are often described as “living drugs” and possess totally different pharmacokinetics compared to classical small molecules, and therefore even the regulatory agencies are not streamlined for assessments of such products.

Hence, the transition from bench to the clinic and accordingly for industry toward acquiring marketing authorization will require collaboration between different disciplines including researchers, physicians, industrial stakeholders, regulatory agencies, and policy makers.

## Discussion—Future Perspectives

The development of therapeutic approaches based on genome editing by designer nucleases is proceeding with great speed and utilizes as a foundation knowledge produced from decades of traditional gene therapy research. However, any new curative scheme faces new challenges many of which are not foreseen particularly by research labs developing the proof of principle for these important new modalities.

The first perspective under discussion for the entire progress of the field is the actual location at which the therapy will take place. Currently, there are two different models that serve this cause: The centralized model assumes collection of the initial cell product from the patient at a local hospital, shipment of this product to a centralized facility in which the genetic engineering takes place, followed by freezing of the cell therapy product and shipment back to the original location. Thereby, the administering physician thaws the cell engineered product and reinfuses it to the patient. There are several advantages as well as disadvantages with this approach. First, centralized manufacturing is much more familiar with the existing mentality of both regulatory agencies as well as policy makers and governmental or societal stakeholders. However, there are serious limitations: This manufacturing model is intended for products with long shelf life and low degree of personalization, which obviously are not applicable for cell and gene therapy products for which transport can have a profound effect on the underlying biology of the cells of interest. Moreover, there is a high risk of incurring issues related from the distance of the user both geographically and in terms of responsiveness to end user requirements and logistics might face the serious issue of biological waste generation. Generally, the centralized model creates opportunities for errors and mistiming of the cell product delivery.

On the other hand, decentralized manufacturing assumes cell collection and processing locally. Equivalent approaches are currently being employed by hospitals in the context of blood transfusion and transplantation of HSCs. This manufacturing model also has pros and cons: the main advantage of this approach is the general flexibility brought about by being closer to the end user, therefore providing responsiveness to evolving requirements and greater personalization according to patient needs. The area of HSC transplantation has contributed enormously to the progress of the gene therapy field, and from that aspect, the decentralized model is closer to the mentality of tissue transplants, a medical routine since 1975 (Dunbar et al., 2018) and shares a lot of common challenges. However, most of these products are under specific tissue or transplant regulations, and these regulations have debatable applicability on gene therapy products. A key limitation to the decentralized model is exactly one of its assets: the flexibility. For such a manufacturing process to be successful from every possible aspect, it is of critical importance to demonstrate robustness. Therefore, a key question is how it is possible to simultaneously be robust and flexible, specifically taking into account that decentralized manufacturing is based on the expertise and skills of each specialized personnel undertaking the manufacturing in different locations. Another most obvious consideration is the starting material and the variability associated with it. Moreover, the type of culture, the differences in the cultivation media and cytokines used, and the timing of the culture generate additional fluctuations. One plausible approach to decrease user variability or bias would be to apply automation during the manufacturing preferably by closed systems with minimal user interaction. This mentality, ideally could be adopted even from early developments in research labs, suggesting that it would be of great benefit to the field if the cell product was produced already under mock-GMP conditions utilizing automated closed systems and GMP-like grade of media and cytokines. A process of this kind would provide a higher degree of maturity of the cell product and the only open variable step would be the starting material. It should be emphasized that once researchers streamline their processes, they should take into consideration that transfer of a research grade manufacturing to a GMP-like manufacturing would include specific documentation from media and cytokine providers, from retroviral vector providers, and from manufacturers of plasmids or RNPs in the case of genome editing. Also, it is generally advisable to utilize one module in the automation step and not different modules, because the regulatory authorities will ask for specific documentation and accreditation from every single module. Therefore, semi-automation will only create delays during any upcoming evaluation from a regulatory agency compared to full automation. Finally, researchers should keep in mind early on that fetal bovine serum, a material widely used in cell and tissue culture, is not characterized as GMP and therefore it would be eliminated from any future step in the process, requiring optimization of the whole process from the beginning.

As a last remark, successful decentralization would most probably require a new set of highly skilled personnel, possibly creating “technology transfer champions” (Harrison et al., 2018) from the current pool of researchers or students and most importantly students of medical sciences who are young, motivated, and eager to undertake the transition between manufacturing and practice in translational medicine. Additionally, centralized managed control standards and certified operators who receive mandatory re-training and licensing of remote site operations should be seriously considered by the universities, the industry, the government, and the society in general.

## Concluding Remarks

The medical field is surely evolving fast and toward the direction of treating diseases previously incurable by the use of genetic manipulations in the form of classical gene therapy by gene addition but also with the advent of designer nucleases by genome editing. Over the past 20 years, significant milestones have been reached in terms of marketing authorization of gene therapy products and real benefit for a large number of patients has been established. However, the field is still in an immature phase, indicating its huge potential for future growth. To that end, researchers should focus early on toward generating true innovative solutions for patients that have the potential to transfer under GMP conditions and are also comparable price wise to the current state of the art. Super expensive solutions, albeit truly innovative in nature, will most certainly face challenges toward achieving proper reimbursement, thereby jeopardizing their eventual availability to patients. It should be emphasized that adoption of poor organization strategies and lack of risk mitigation measures early in the development has the potential to undermine the future success of an otherwise promising strategy or product, specifically in the area of genome editing. If such strategies are adopted early on from researchers, it is possible that previously unforeseen or unanticipated obstacles on the path to approval, often taking decades to address, will be omitted, increasing the wider applicability of genetic therapies, and unlocking their true potential.

## Author Contributions

EP researched the literature and wrote the manuscript. AB read the manuscript, provided feedback and final approval. Both authors contributed to the article and approved the submitted version.

## Conflict of Interest

AB and EP are employees of Miltenyi Biotec.

## Footnotes

^1^ https://www.sciencehistory.org/distillations/the-death-of-jesse-gelsinger-20-years-later
